# Identification and Characterization of *Ixodes scapularis* Antigens That Elicit Tick Immunity Using Yeast Surface Display

**DOI:** 10.1371/journal.pone.0015926

**Published:** 2011-01-05

**Authors:** Tim J. Schuijt, Sukanya Narasimhan, Sirlei Daffre, Kathleen DePonte, Joppe W. R. Hovius, Cornelis van't Veer, Tom van der Poll, Kamran Bakhtiari, Joost C. M. Meijers, Eric T. Boder, Alje P. van Dam, Erol Fikrig

**Affiliations:** 1 Department of Internal Medicine, Yale University School of Medicine, New Haven, Connecticut, United States of America; 2 Center for Experimental and Molecular Medicine, Academic Medical Center, University of Amsterdam, Amsterdam, The Netherlands; 3 Department of Experimental Vascular Medicine, Academic Medical Center, University of Amsterdam, Amsterdam, The Netherlands; 4 Department of Medical Microbiology, Leiden University Medical Center, Leiden, The Netherlands; 5 Departamento de Parasitologia, Universidade de São Paulo, São Paulo, Brazil; 6 Department of Chemical and Biomolecular of Engineering, University of Tennessee, Knoxville, Tennessee, United States of America; 7 Department of Medical Microbiology, Onze Lieve Vrouwe Gasthuis, Amsterdam, The Netherlands; New York University School of Medicine, United States of America

## Abstract

Repeated exposure of rabbits and other animals to ticks results in acquired resistance or immunity to subsequent tick bites and is partially elicited by antibodies directed against tick antigens. In this study we demonstrate the utility of a yeast surface display approach to identify tick salivary antigens that react with tick-immune serum. We constructed an *Ixodes scapularis* nymphal salivary gland yeast surface display library and screened the library with nymph-immune rabbit sera and identified five salivary antigens. Four of these proteins, designated P8, P19, P23 and P32, had a predicted signal sequence. We generated recombinant (r) P8, P19 and P23 in a *Drosophila* expression system for functional and immunization studies. rP8 showed anti-complement activity and rP23 demonstrated anti-coagulant activity. *Ixodes scapularis* feeding was significantly impaired when nymphs were fed on rabbits immunized with a cocktail of rP8, rP19 and rP23, a hall mark of tick-immunity. These studies also suggest that these antigens may serve as potential vaccine candidates to thwart tick feeding.

## Introduction

*Ixodes scapularis* and *Ixodes ricinus* ticks transmit pathogens such as *Borrelia, Babesia, Anaplasma* and selected flaviviruses [1]. In order to acquire a successful blood meal, these ticks engorge for several days on a mammalian host and counter the haemostatic system and immune responses of the host by spitting tick saliva into the skin [2]. Tick saliva contains proteins that inhibit T-cells [3], B-cells [4], the complement system [5], [6], [7], [8], dendritic cells [9] and the coagulation system [10], [11], [12], [13]. Even though ticks modulate and dampen host immune responses to ensure successful feeding, upon repeated tick infestations some animals develop an immune response resulting in tick rejection. This phenomenon, referred to as ‘tick immunity’, was first described by William Trager in 1939, when he observed that *Dermacentor variabilis* ticks were not able to efficiently engorge on guinea pigs that had previously been exposed to several tick infestations [14]. Parameters of tick-immunity include decreased numbers of ticks feeding on the host, delayed time of engorgement, a reduction in tick weight, the inability to molt and decreased fecundity. Mast cells, basophils, eosinophils [15], and antibodies [16] against exposed and concealed [17] tick proteins play a role in tick-immunity.

In contrast to animals such as guinea pigs and rabbits, mice, do not develop the hall marks of tick-immunity upon repeated infestations with *Ixodes scapularis* ticks [18]. The mechanism underlying this difference remains to be understood. However, immune responses directed against tick proteins was shown to reduce *Borrelia* transmission when infected ticks fed on mice that were repeatedly infested with ticks [18]. *Borrelia* transmission in mice passively administered serum from tick-immune rabbits was also reduced when challenged with *Borrelia burgdorferi*-infected *I. scapularis* nymphs [19]. These observations uncouple tick feeding from pathogen transmission and suggest that while the tick-immune serum is unable to thwart tick feeding in mice, tick-immune serum contains antibodies directed against tick salivary proteins critical for *Borrelia* transmission to mice. Repeated exposure to tick bites is also associated with fewer episodes of Lyme disease in residents living in areas where *B. burgdorferi* infection is endemic [20]. Therefore, identification of tick salivary antigens that react with tick-immune serum would provide the preamble for a molecular understanding of tick feeding as well as pathogen transmission and also provide novel vaccine targets both to block tick feeding and pathogen transmission [21].

Immunoscreening of cDNA expression libraries using a phage display approach has identified several tick salivary proteins that react with tick-immune serum [22], [23]. A limitation with phage-displayed proteins is that they lack eukaryotic post-translational modifications that might contribute to critical epitopes, and preclude the identification of such antigens by phage display screening. Therefore, additional screening efforts that exploit novel high-throughput approaches would be essential to generate a comprehensive array of salivary antigens that react with tick-immune sera. Such a detailed catalog would help develop and distill a critical subset of tick salivary antigens that might serve as vaccines to block tick feeding and impair pathogen transmission. Towards this goal, we adapted the Yeast Surface Display (YSD) approach [24], that allows eukaryotic proteins to be displayed in a near-native form [25]. While YSD has been traditionally applied to study protein-protein interactions, we have in this report utilized the YSD approach to identify a subset of salivary proteins from *I. scapularis* nymphal stage that react with nymph-immune rabbit sera.

## Results

### Identification of antigenic *I. scapularis* salivary proteins from the nymphal stage

**Figure 1:**
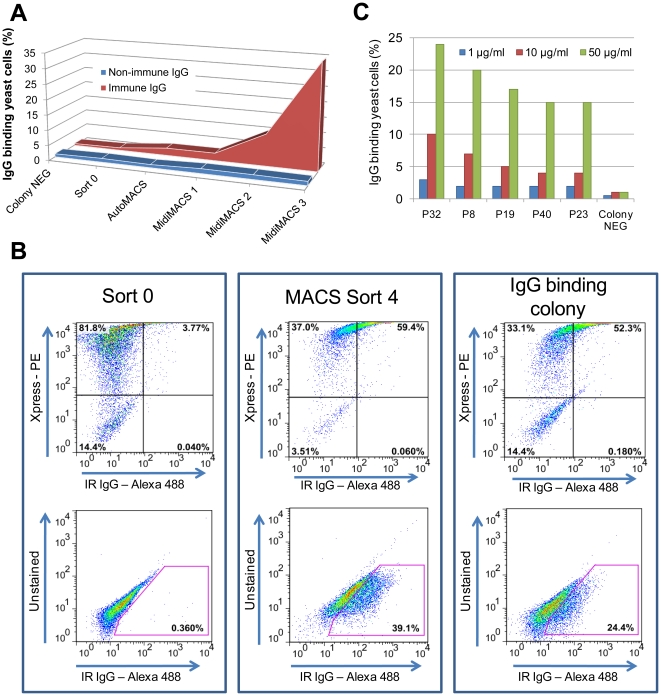
Enrichment and selection of yeast cells expressing immunogenic *I. scapularis* salivary proteins. FACS analysis of yeast cells using nymph-immune rabbit IgG (red) and IgG derived from normal rabbit serum (blue) of transformed yeast cells (sort 0); autoMACS sort (AutoMACS); MidiMACS sort 1, 2 and 3 (MidiMACS1, 2 and 3). (B) Selection for IgG binding clones using 50 µg/ml Alexa-488 conjugated nymph immune rabbit IgG. Sort 0 was used as a negative control and MACS sort 4 as a positive control. Upper panel: mouse anti-Xpress antibody binding to induced yeast cells (PE). Lower panel: Cells binding nymph immune rabbit IgG (IR IgG) shown within the pink gate (Alexa-488). (C) Titration of binding of each unique using 50 µg/ml (green bars), 10 µg/ml (red bars) or 1 µg/ml (blue bars) of immune rabbit IgG. The percentage IgG binding yeast cells were determined by FACS analysis as was shown in the right lower panel of Fig. 1B.

A YSD expression library of *I. scapularis* salivary gland cDNAs was probed with purified IgG from pooled sera from nymph-immune rabbits. After 4 rounds of magnetic-activated cell sorting (MACS) screen, a ∼110-fold enrichment of yeast cells expressing salivary proteins recognized by rabbit nymph-immune serum (**Fig. 1** ***A,B***) was obtained. The library sorted with IgG purified from non-immune serum did not show binding and did not provide enrichment of yeast cells (**Fig. 1** ***A***). Two hundred fifty colonies from the 4^th^ sort were individually tested for their ability to bind to rabbit nymph-immune antiserum by fluorescence-activated cell sorting (FACS) analysis. Recombinant plasmids were isolated from 98 positive colonies and insert sizes compared by restriction digestion analysis. Clones with similar insert sizes were grouped and representative clones sequenced. Five unique clones encoding tick salivary proteins were identified and provided a unique identifier based on their putative molecular mass as shown in **Table 1**. YSD was also useful in comparing the specificity and avidity of the antigen-antibody interaction between different clones using varying amounts of IgG from immune rabbits (**Fig. 1** ***C***). *In silico* analysis of P23 and P32 protein sequences revealed homology with putative secreted salivary gland proteins of *I. scapularis*, P19 was found to share homology with proteins from *Rhipicephalus annulatus* and *Heamaphysalis qinghaiensis* [26], [27]. P8 showed similarities with the *I. scapularis* anticoagulant protein, Salp14, and the putative anticoagulant Salp9pac. P40 was identical to *I. scapularis* Transducing (beta)-like 2 protein (**Table 1**).

**Table 1:** Antigenic *Ixodes scapularis* salivary proteins identified by immunoscreening a nymphal *Ixodes scapularis* yeast display library using nymph-immune rabbit serum.

Positive colonies	GenbankAccession Number	Match to NR protein database#	ORF	MW* (kDa)	pI*	Domains	Paralogues†	Signal sequence‡
P8	HQ605983	*I. scapularis,* Salp14GenBank: AAK97824.1E value 2e^−43^ *I. scapularis*, Salp9pacGenBank: AAN03859.1E value 5e^−48^	276-bp	7.9	4.3	NO	YES	YES
P19	XM_002399589.1	*Rhipicephalus annulatus*, Ba05GenBank: ABV53333.1E value 6e^−78^ *Haemaphysalis qinghaiensis*,Hq05GenBank AAX37829.1E value 1e^−69^	564-bp	18.7	5.8	NO	NO	YES
P23	HQ605984	*I. scapularis,* putative secreted SG peptide, GenBank: XP_002405271.1E value 4e^−37^	669-bp	22.7	9.5	NO	YES	YES
P32	HM802761.1	*I. scapularis,* putative secreted SG peptide, GenBank: AAV80775.1E value 3e^−40^	912-bp	31.7	5.6	NO	NO	YES
P40	HM802762.1	*I. scapularis* Transducing (beta)-like 2 (Tbl2) protein,GenBank: XM_002416416.1E value 9e^−140^	1089-bp	40.1	7.2	WD40(NCBI CDD:cl02567)	NO	NO

#Homology search performed using BLAST (www.ncbi.nlm.nih.gov/BLAST).

*Theoretical molecular weight (MW) and isoelectric point (pI) using ExPASy proteomics server (http://www.expasy.ch/tools/pi_tool.html).

† Proteins ≤40% identity using the database of VectorBASE (http://iscapularis.vectorbase.org/) and the GenBank database (www.ncbi.nlm.nih.gov/blast/Blast.cgi).

‡ Secretory signal sequence as assessed by the SignalP 3.0 signal prediction server (www.cbs.dtu.dk/services/SignalP/).

**Figure 2:**
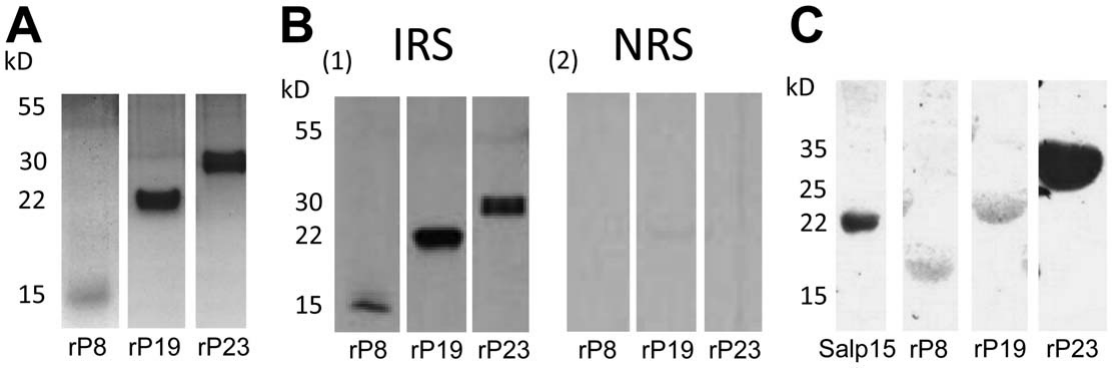
Purified recombinant *Ixodes scapularis* salivary proteins. (A) Coomassie blue staining of purified recombinant *I. scapularis* salivary proteins rP8, rP19 and rP23 electrophoresed on SDS 12% polyacrylamide gel. (B) Western blot analysis of the recombinant proteins probed with nymph-immune rabbit serum (IRS) and normal rabbit serum (NRS). (C) PAS staining of rP8, rP19, rP23 and Salp15 electrophoresed on SDS 12% polyacrylamide gel.

With the exception of P40, all other antigens identified contained a canonical secretory signal sequence. With an initial focus on secreted salivary proteins, we chose to exclude P40 in this study. P8, P19 and P23 were produced as recombinant proteins in a *Drosophila* expression system and purified using Ni-NTA Superflow chromatography (**Fig. 2** ***A***). *Escherichia coli* DH5α cells transformed with the pMT/Bip/V5-HisA plasmid containing *p32* were not viable, which precluded plasmid generation for transfection of S2 cells and rP32 protein production. The three recombinant salivary proteins were recognized avidly by antibodies in nymph-immune rabbit serum (**Fig. 2** ***B*** **, panel 1**), and did not react with normal rabbit serum (**Fig. 2** ***B*** **, panel 2**). Analysis of the amino acid sequences of the proteins (www.cbs.dtu.dk/services/NetNGlyc/) revealed that P8 and P19 have 1 predicted *N*-glycosylation site and P23 has 3 predicted *N*-glycosylation sites. Indeed, consistent with the *in silico* glycosylation predictions, glycoprotein staining with periodic acid-Schiff (PAS) indicated that rP8 and rP19 were glycosylated, albeit to a lesser extent compared to rP23 (**Fig. 2** ***C***).

### Expression of the *p19*, *p23*, *p32* and *p8* genes in different *I. scapularis* stages

**Figure 3:**
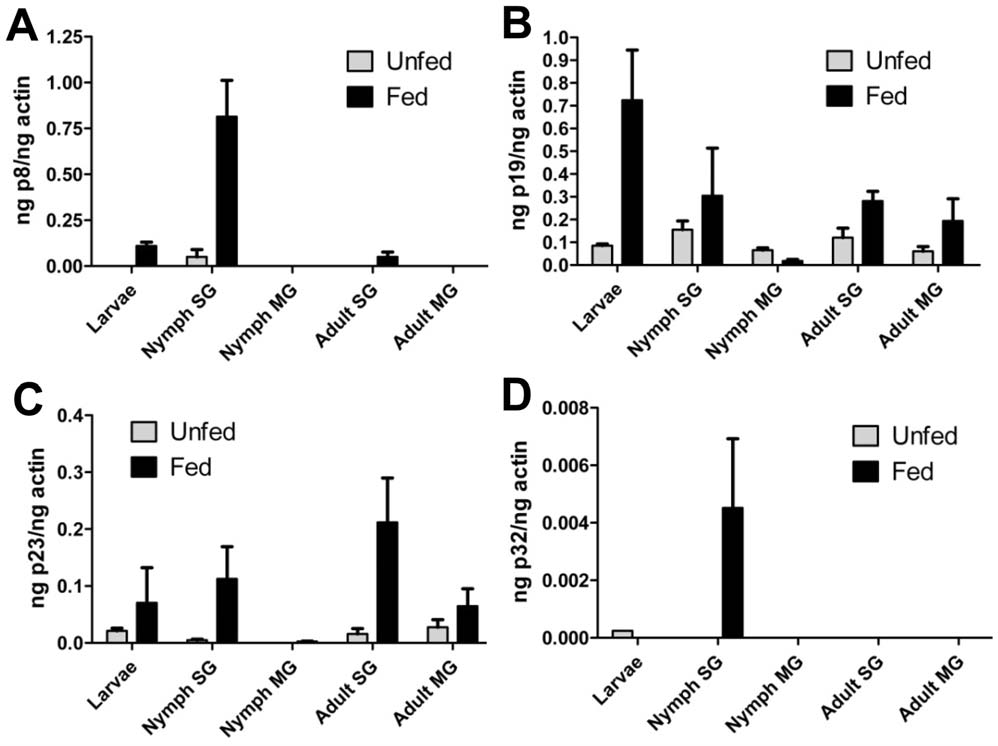
Expression of the genes coding for the four salivary proteins during several life stages of *I. scapularis*. Quantitative reverse-transcription polymerase chain reaction performed on RNA isolated from whole larvae, and from salivary glands and midguts *I. scapularis* from nymphs and adults. Expression profile of: A. *p8*; B. *p19*; C. *p23*; and D. *p32* in unfed (grey bars) and fed ticks (black bars). SG; salivary glands, MG; midguts.

*p19* and *p23* were expressed in larval, nymphal and adult ticks, while *p32* and *p8* were primarily expressed in nymphs (**Fig. 3**). As expected all 4 genes were expressed in tick salivary glands and significantly induced upon feeding. In addition, *p19* and *p23* showed additional expression in the tick gut in selected developmental stages and *p8* and *p32* were preferentially expressed in the nymphal salivary glands.

### rP8 protects Borrelia from complement-mediated killing

**Figure 4:**
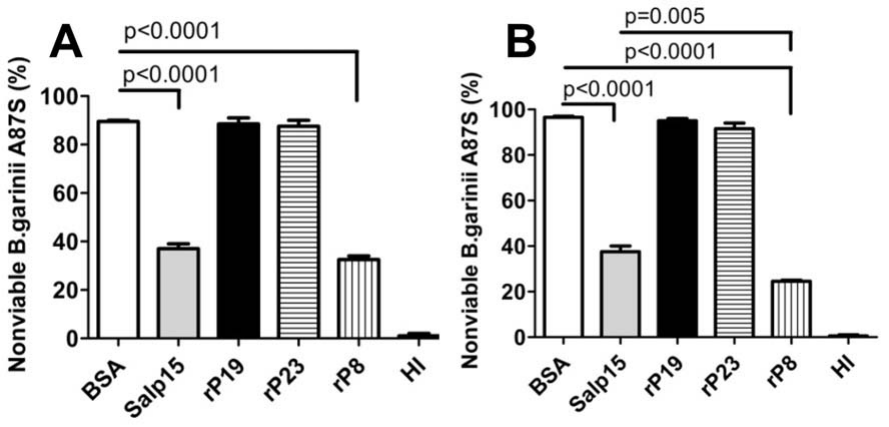
Influence of recombinant salivary proteins on human complement system. Serum sensitive strain *Borrelia garinii* A87S was incubated with 12.5% NHS in the presence of BSA, Salp15, rP19, rP23 or rP8 for (A) 1.5 hours; or (B) 4.5 hours and the percentage of immotile spirochetes were determined. Control spirochetes were incubated with heat-inactivated NHS (HI). Two hundred spirochetes were counted. Results represent mean ± SEM of values from a representative of 3 replicate experiments.

Since no functional domains were found by *in silico* analysis, we examined each of the three recombinant proteins in assays to test for predominant biochemical activities represented in tick saliva, i.e. anticomplement [6], [8], [28] and anticoagulant activity [10], [11], [12], [13], [29], [30]. Unlike, *B. burgdorferi* sensu stricto, *B. garinii* is a human complement sensitive strain [31]. Therefore, we utilized a *B. garinii* killing assay [8] to investigate whether rP8, rP19 and rP23 were able to protect spirochetes from or inhibit the human complement system. rP8 significantly reduced complement-mediated killing of *B. garinii* A87S (**Fig. 4**) after 1.5 hours (**Fig. 4** ***A***; 90±0.9% for BSA versus 31±1.2% for rP8, p<0.0001) and 4.5 hours (**Fig. 4** ***B***; 97±0.6% for BSA versus 25±0.9% for rP8, p<0.0001), in a dose dependent manner. *B. garinii* spirochetes incubated with heat-inactivated NHS remained viable at all time points examined (**Fig. 4**). None of the other recombinant proteins provided protection against complement-mediated killing (**Fig. 4**). The *I. scapularis* salivary protein Salp15 was used as a positive control [8].

### rP23 inhibits coagulation of human plasma

**Figure 5:**
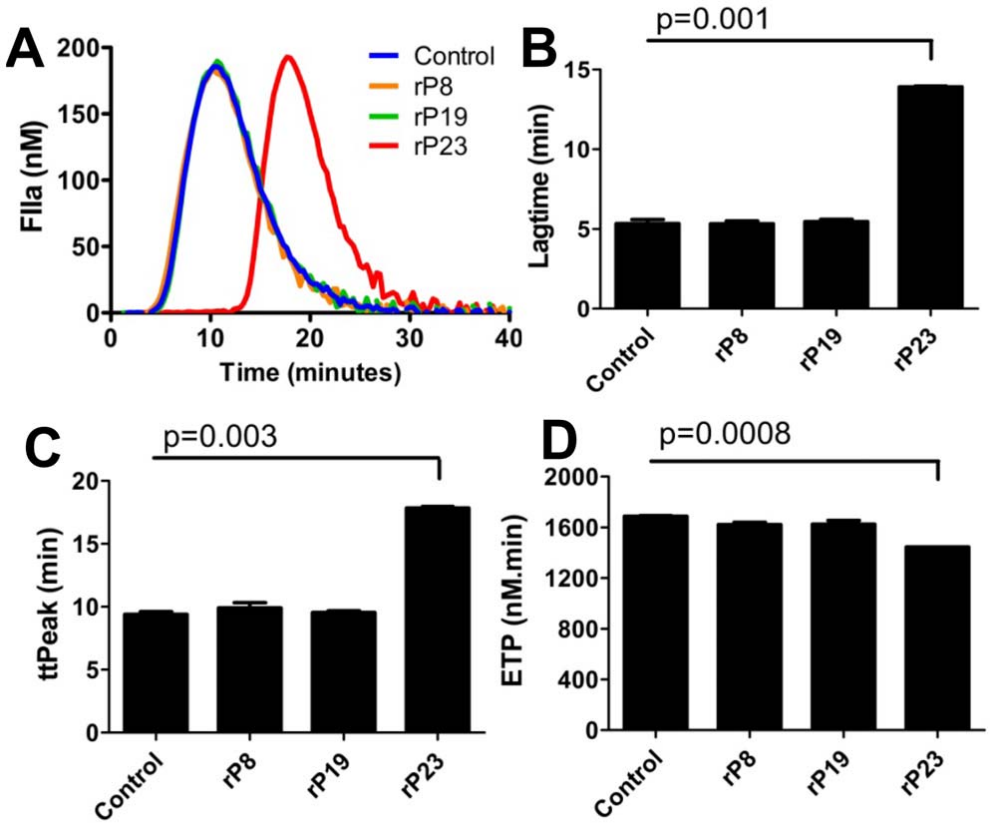
Influence of recombinant salivary proteins on human coagulation system. (A) Thrombin generation was initiated in human pooled normal plasma with 1 pM tissue factor (TF) in the presence of rP8 (orange), rP19 (green) or rP23 (red) and thrombin generation was measured using a fluorogenic substrate. (B) Lagtime, (C) time to peak (ttpeak) and (D) Endogenous Thrombin Potential (ETP) were measured. Unpaired t-test was used to determine statistical significance. Representatives of three experiments are shown. Results described represent the mean ± SEM.

Calibrated automated thrombography (CAT) was used to assess the effect of the recombinant *I. scapularis* salivary proteins on tissue factor initiated thrombin generation. In normal human pooled plasma, recombinant rP23 delayed thrombin generation (**Fig. 5** ***A***), with significant prolongation of lag time and time to peak (**Fig. 5** ***B,C***) in a dose dependent manner, suggesting that rP23 may influence the initiation phase of coagulation. In addition, determination of the total amount of thrombin formed (Endogenous Thrombin Potential, ETP) showed that rP23 significantly reduced thrombin generation by 18% compared to ETP in the absence of rP23 (**Fig. 5** ***D***). rP8 and rP19 at similar concentrations did not affect thrombin generation (**Fig. 5**).

### Immunization with recombinant P8, P19 and P23 impairs nymphal tick feeding on rabbits

**Figure 6:**
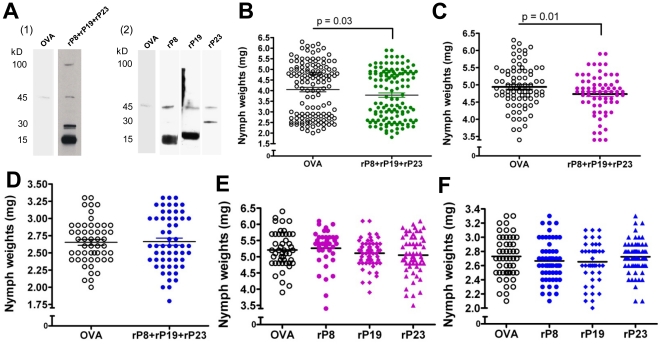
Nymph feeding after rabbit immunization with recombinant salivary proteins. (A) Nymph salivary gland extract probed with rP8/rP19/rP23 immune rabbit serum (panel 1), rP8 or rP19 or rP23 immune rabbit serum (panel 2), and with serum from the control (OVA) rabbit. (B) Nymph weights recovered from the control and the rP8/rP19/rP23 immunized rabbits. Tick weights of the heavy group of nymphs (C) and light group of nymphs (D) fed on rP8/rP19/rP23 immunized rabbits compared to the control rabbits. Weights of the heavy group of nymphs (E) and light group (F) of nymphs fed on rabbits immunized singly with OVA, rP19, rP8 or rP23 respectively. The horizontal bars represent the means of the respective groups. Unpaired t-test was used to determine statistical significance.

Three rabbits were immunized with a cocktail of rP8, rP19 and rP23. Nymphal salivary gland extract (SGE) probed with immune sera from each of the rabbits recognized the native proteins (**Fig. 6** ***A*** **, panel 1**). A 45 kDa band appeared in all blots most likely due to binding of anti-rabbit IgG to host globulins present in the fed SGE [32]. Immune sera from 3 control rabbits immunized with OVA did not react with proteins in the nymphal SGE (**Fig. 6** ***A*** **, panel 1**). Upon challenge of the immunized rabbits with *I. scapularis* nymphs, comparable numbers of nymphs fed to repletion on the control and experimental animals (127 nymphs on the immunized group versus 137 on the control group). Nymphs feeding on rabbits immunized with the cocktail of rP8, rP19 and rP23 were significantly lighter than nymphs feeding on the OVA control rabbits (**Fig. 6** ***B***; 3.7±0.1 mg and 4.0±0.1 mg, respectively, p = 0.03). An independent control experiment showed that, after feeding on normal rabbits, nymphs with a weight of 3.3 mg and below consistently molted into males, while the heavy group of nymphs (3.4 mg and above) molted into female adult ticks (**Table 2**). Therefore, engorged ticks were also divided into subgroups (light and heavy) (**Fig. 6** ***C–D***), Analysis of the subgroups showed a significant reduction in engorgement weights in the heavy group of nymphs fed on the rP8, rP19 and rP23 cocktail immunized rabbits compared to the heavy group of nymphs fed on control rabbits (4.7±0.1 mg and 4.9±0.1 mg, p = 0.01) (**Fig. 6** ***C***). There was no significant difference between the light group of nymphs feeding on immunized rabbits versus controls (**Fig. 6** ***D***). Finally, when three rabbits per group were immunized with single proteins, nymphal SGE probed with immune sera from each of the rabbits recognized the native proteins (**Fig. 6** ***A*** **, panel 2**), but there was no difference in nymph weights, compared to the control OVA-immunized animals in both heavy (**Fig. 6** ***E***) and light groups (**Fig. 6** ***F***).

**Table 2:** Relationship between post-engorgement weights and resultant sexes in *Ixodes scapularis* nymphs.

Nymphal Engorgement Weights	Males	Females
2.0–3.3 mg	92	0
3.4–6.2 mg	0	110

## Discussion

*Ixodes* ticks transmit numerous pathogens, including bacteria, protozoa and selected flaviviruses [33]. Anti-tick vaccines could potentially prevent transmission of *Borrelia*, as well as other pathogens, from the tick to the host. Acquired resistance to tick feeding can also impair pathogen transmission [34], [35]. Several studies have shown a critical role for the humoral response in tick immunity [16], [36] and provided the impetus to identify antigens that elicit tick-immunity by exploiting approaches including immunoscreening of cDNA expression libraries generated in routine prokaryotic expression systems [22], [23]. However, the prokaryotic systems are not capable of displaying the antigens with eukaryotic post-translational modifications. When these modifications are critical determinants of antigenic epitopes, prokaryotic expression systems preclude identification of such antigens. It is therefore critical to exploit additional strategies to circumvent these limitations. It is also important to note that the proteome of the developmental stages of ticks are likely different [37] and antigens critical for one stage might not necessarily be critical for all stages. Therefore, it is important to develop viable and high-throughput strategies to screen for salivary antigens that react with stage-specific immune sera. This would help build a comprehensive catalog of salivary antigens that react with tick-immune serum against different developmental stages of *Ixodes scapularis*, and facilitate the development of functional vaccines targeting critical developmental stages. Since, *I. scapularis* nymphs are critical for disease transmission to humans [38], in this study we advance our understanding of nymphal salivary proteins that react with nymph-immune rabbit sera. It is expected that these antigens might play a role in tick feeding and immunity against these antigens might impair tick feeding. Such a phenotype would therefore also affect the success of nymphal molting to adults and would be conductive to control tick populations. Decreased feeding might also lead to decreased pathogen transmission. Additionally, these antigens might provide functions critical for pathogen transmission [6], [8], [39]. Therefore, we used the YSD approach, to identify salivary antigens that react with nymph-immune serum and examine their role in the context of nymphal feeding success.

We demonstrate that YSD technology is a robust tool for the identification of antigenic salivary gland proteins. The major advantage of YSD over other expression systems such as phage display, is that yeast cells have eukaryotic machinery allowing the display of eukaryotic proteins with post-translational modifications like glycosylation, phosphorylation and correct disulphide bond formation [40] and hence, utilized for several applications including protein affinity maturation, epitope mapping, and cell adhesion molecule engineering [25], [41]. To date, there are only a few reports describing the utility of YSD for cDNA library screening and have addressed human cDNA libraries [42], [43]. This study extends the utility of YSD to perform high throughput immunoscreening of a YSD library of nymphal *I. scapularis* salivary gland cDNAs to enrich for yeast cells expressing antigenic tick proteins (**Fig. 1**).

The YSD library of *I. scapularis* salivary gland cDNA was prepared from RNA isolated from replete nymphs. We are cognizant that the salivary gland proteome is dynamic, and the protein profiles change during feeding. The current screening effort is therefore not likely to identify antigens expressed specifically at early time points of feeding. Five novel antigenic tick salivary proteins were identified using the YSD approach (**Table 1**). While P23 and P32 only showed homology with putative secreted salivary gland proteins, with no known functions. P8 showed homology with the putative anticoagulant Salp9pac and anticoagulant Salp14 family of proteins. Salp14 was identified earlier by Das *et al* [22] by immunoscreening a phage expression library of *I.scapularis* nymphal cDNAs using tick-immune sera [10]. P19 was homologous to a larval immunogenic protein, Ba05 from *Rhipicephalus annulatus* [27] and the protein Hq05 from *Haemaphysalis qinghaiensis* [26] (93 and 82% identity respectively). Expression analysis of *Hq05* showed expression specifically in salivary glands of nymphs and adults, but not in eggs and larvae of *H. qinghaiensis* [26]. P40 showed similarities with a putative G-protein from *I. scapularis* (XP_002416461.1) and with Transducin beta-like 2 proteins (NP_001008084) from several species including mammals. P40 contains 7 copies of the WD40 domain, which is found in several eukaryotic proteins that are involved in diverse functions including pre-mRNA processing, signal transduction, cytoskeleton assembly and cell cycle control [44], [45], [46]. P40 lacked a canonical secretory signal sequence and possibly not secreted in tick saliva. However, based on the reactivity of P40 with tick-immune sera (**Table 1**), we speculate that P40 might be secreted in tick saliva by other/novel secretory mechanisms and warrants detailed verification and therefore not included in this study. Four of the five identified proteins, i.e. P8, P19, P23 and P32, had a predicted secretory signal sequence as assessed by the SignalP 3.0 signal prediction server (www.cbs.dtu.dk/services/SignalP/) and were therefore selected for further analysis. As expected, the identified genes *p8, p19, p23* and *p32* encoding for the antigenic tick proteins were all induced upon nymphal feeding (**Fig. 3**). While *p19* and *p23* were expressed both during larval and adult stages, *p8* and *p32* were preferentially expressed in the nymphal stage, indicating that some salivary proteins might play a stage-specific role. Next, recombinant P8, P19 and P23 were made in S2 cells using a *Drosophila* expression system (**Fig. 2**). Attempts to generate stable S2 cell lines expressing rP32 were unsuccessful. All three recombinant proteins showed glycosylations as seen by a positive reaction using the Periodic Acid Schiff method (**Fig. 2** ***C***), suggesting that these proteins might also be glycosylated in *I. scapularis in vivo* and might account for the slightly increased molecular masses of the native P8 and P23 (**Fig. 6** ***A***). *In silico* analysis of the proteins for potential glycosylation sites using the NetNGlyc server (www.cbs.dtu.dk/services/NetNGlyc/) also suggested one and 3 potential N-glycosylation sites on P8 and P23, respectively.

Despite the homology between P8 and the anticoagulant Salp14 [10], [47], rP8 did not have any effect on thrombin formation in human plasma. We have previously shown that Salp9pac, also homologous to Salp14, did not have anticoagulant properties [10]. The anticoagulant Salp14 has a positively charged stretch of 20 amino acids at the C-terminal tail compared to rP8 and Salp9pac, which is most likely important for interaction with the coagulant proteins [10]. rP23 demonstrated anticoagulant activity and significantly delayed thrombin generation in human plasma (**Fig. 5**). Interestingly, P23 does not contain any known anti-coagulant domains and might represent a novel type of anticoagulant. The mechanism of anticoagulation by rP23 remains to be determined.

To investigate the three proteins for anti-complement activity we used a *B. garinii* killing assay, since *B. garinii* is a serum sensitive strain compared to *B. burgdorferi* sensu stricto [8], [31]. We have previously demonstrated that the serum-sensitive *B. garinii* A87S strain is killed by the human complement system after formation of the C5b-9 membrane attack complex and that *Ixodes* Salp15 provided protection against this complement-mediated killing [8]. When *Borrelia* was incubated with normal human serum, rP8 was also able to protect *Borrelia* against complement-mediated killing (**Fig. 4**). Whether, rP8 provides this protection by virtue of an anticomplement activity or by physically binding to spirochetes and shielding the spirochetes from complement attack remains to be examined.

We next examined if immunity against these recombinant salivary proteins would recapitulate tick immunity in rabbits. When rabbits were immunized with a cocktail of rP8, rP19 and rP23 and challenged with pathogen-free nymphs, feeding efficiency was modestly, but significantly, decreased compared to nymphs that fed on OVA-immunized rabbits, as determined by engorgement weights (**Fig. 6** ***B***). Previous experiments in our laboratory, consistent with observations by Hu et al [48], have shown that the heavy group of nymphs (3.4 mg and higher) consistently molted into adult females, while the light group of nymphs (3.3 mg and lighter) molted into adult males (**Table 2**). The impaired feeding phenotype was significant in the to-be-female group (**Fig. 6** ***C***), since when fed nymphs were separated into heavy (to-be-female) and light (to-be-male) groups of nymphs, no difference in post-engorgement weights was found in the to-be male group of nymphs fed on the rP8, rP19, rP23 immunized rabbits compared to controls (**Fig. 6** ***D***). In addition, two weight populations were seen in the to-be male group of nymphs fed on the immunized animals (**Fig. 6** ***D***), possibly due to to-be female ticks that showed impaired feeding due to immunization with the recombinant tick proteins controls. It is possible that the to-be male group of ticks differentially express salivary proteins while feeding on the host compared to the to-be female group of nymphs. P8, P19 and/or P23 might play a redundant role in feeding in the to-be male nymphs. Future studies will determine whether the cocktail vaccine (rP8, rP19 and rP23) might also impair pathogen transmission from the tick to the host.

In conclusion, these data show that cocktail immunization targeting multiple (functional) salivary proteins resulted in impaired tick feeding, while immunizations with individual proteins did not have an effect. It is conceivable that targeting 3 antigens simultaneously provided neutralization of antigens important for suppressing several arms of the host defense (in this case, host coagulation and complement) and resulted in impaired tick feeding. Further, the past decade has demonstrated that the tick transcriptome elaborates an array of functional paralogs [49]. Several anticomplement [6], [8], [28] and anticoagulant proteins [10], [11], [12], [13], [29], [30] have been described and characterized in *Ixodes* ticks. This functional redundancy is central to the tick's ability to feed successfully. To efficiently block tick feeding, it might be important to immunize animals with cocktails of several anticomplement or anticoagulant proteins to circumvent fall-back strategies of the tick. Since tick-immunity effectively disables the ability of the tick to feed, presumably, tick-immune serum targets the critical subsets of functional paralogs. Thus, intensive screening using the YSD approach would provide a comprehensive list of antigens that react with tick-immune serum. This would enable us to group subsets of functional paralogs critical for feeding and facilitate the development of an effective cocktail vaccine to block tick feeding.

## Materials and Methods

### Ticks and animals

*I. scapularis* adults, nymphs and larvae were obtained from a tick colony at the Connecticut Agricultural Experiment Station in New Haven CT, USA. Ticks were maintained at 23°C and 85% relative humidity under a 14 hour light, 10 hour dark photoperiod. For the immunization studies, 6 week old inbred New Zealand white rabbits (Charles River Laboratories) were used. The work reported in this study is fully compliant with and approved by institutional policies pertinent to biosafety and animal care protocols. The protocol for the use of mice and rabbits was reviewed and approved by the Yale Animal Care and Use Committee (protocol number 2008-07941, approval date is 03/31/10 to 3/31/11).

### Construction of an *I. scapularis* salivary gland cDNA library

RNA was purified from the salivary glands from 1000 *I. scapularis* nymphs fed to repletion (repletion achieved between 72 and 96 h), and cDNAs directionally cloned into the *EcoR*I and *Not*I sites of the yeast expression vector pYD1 (Invitrogen, CA) to generate a salivary gland expression library wherein that tick salivary proteins were expressed as Aga2 fusion proteins on the yeast surface (Invitrogen, CA). *PstI* digestion of plasmids purified from 24 clones of the pYD1-salivary gland library, showed an average insert size of 2.1 kb and 100% of the clones contained inserts. The unamplified library titre was 0.5×10^6^ cfu/ml. Growth of transformant yeast cells and induction of recombinant protein production was done essentially as detailed by Chao *et al*. [50]. Briefly, fresh *Saccharomyces cerevisiae* EBY100 cells (Invitrogen, CA) with 5 µg of DNA were electroporated and subsequently grown in SDCAA medium (2% dextrose, 0.67% yeast nitrogen base, 0.5% bacto amino acids, 30 mM NaHPO_4_, 62 mM NaH_2_PO_4_). Induction of surface protein expression was done as described below.

### Purification and conjugation of IgG from tick-immune rabbit sera

Two *I. scapularis* nymph-immune rabbits were generated as described earlier [19] and sera tested by western blot analysis to confirm reactivity with nymph SGE. IgG was purified from the nymph-immune rabbit sera using the Melon Gel IgG purification kit (Thermo Fisher Scientific inc, Rockford, IL). IgG concentration was measured using the BCA protein assay kit (Thermo Fisher Scientific inc., Rockford, IL). Rabbit IgG was labeled with Alexa-488 using the Alexa Fluor® 488 Protein Labeling Kit (Invitrogen, CA) according to the manufacturer's protocol.

### Selection of yeast cells expressing immunogenic *I. scapularis* nymph salivary proteins

To induce surface protein expression, approximately, 1×10^10^ transformed yeast cells were grown for 24 hours at 28°C and induced with galactose and selected by 4 rounds of MACS sorting, as described earlier [50]. To induce surface protein expression, approximately, 1×10^10^ transformed yeast cells were grown for 24 hours at 28°C in 100 ml of SGCAA medium (2% galactose, 0.67% yeast nitrogen base, 0.5% bacto amino acids, 30 mM NaHPO_4_, 62 mM NaH_2_PO_4_). After induction with galactose, surface expression was demonstrated by indirect immunostaining with an antibody against the Xpress-epitope located on the N-terminal part of the expressed salivary protein on the yeast cell surface [50]. Ten thousand cells were examined on a FACSCalibur flow cytometer (Beckton Dickinson, Franklin Lakes, NJ) and data analyzed using the FlowJo software (Tree Star, Ashland, OR). The first round of selection was done using AutoMACS^TM^ (Miltenyi Biotec, Auburn, CA). 2×10^10^ transformed yeast cells were washed 3 times with cold MACS (0.5% BSA, 2 mM EDTA) buffer and pelleted at 600 x*g* for 10 minutes. Next, cells were resuspended in cold MACS buffer and incubated with 30 µg/ml of purified nymph-immune rabbit IgG and incubated with gentle rotation for 30 minutes at 4°C. Subsequently, cells were washed 2 times and resuspended in 15 ml MACS buffer. 1 ml of goat-anti rabbit microbeads (Miltenyi Biotec, Auburn, CA) was added and incubated for 15 minutes at 4°C. Cells were washed 3 times, resuspended in 150 ml of MACS buffer and subjected to magnetic separation. The sorted cells were grown in SDCAA medium with Pen/Strep for 24 hours at 30°C. Subsequently, the cells were further selected by 3 rounds of MidiMACS sorting under the same conditions as described above. For screening of individual clones, 1×10^7^ induced yeast cells were incubated on a shaking incubator with 33 µg/µl Alexa-488 conjugated nymph-immune rabbit IgG in MACS buffer for 45 minutes at room temperature. The cells were washed and resuspended in MACS buffer and analyzed on a FACSCalibur flow cytometer as described above. Plasmid DNA was isolated from individual positive clones using the Zymoprep^TM^ II Yeast Plasmid Miniprep kit (Zymo research, Orange, CA), transformed into *E. coli* DH5α (Invitrogen, CA) and plated on LB plates containing 100 µg/ml ampicillin. Plasmid DNA was then isolated from bacterial colonies using the Plasmid Miniprep kit (Qiagen, CA), digested with *Xho*I and *BamH*I (New England Biolabs, MA) to assess insert sizes and unique clones sent for sequencing (Keck Facility, Yale University).

### Production of recombinant salivary proteins

*p8, p19* and *p23* cDNAs were cloned in frame into the pMT/Bip/V5-HisA plasmid containing a His tag, V5 epitope, and a blasticidin resistance gene (Invitrogen, CA), and validated by sequencing. *Drosophila melanogaster* S2 cells were transfected with the plasmids containing *p8, p19* or *p23* and the blasticidin selection vector pCOBlast using the Calcium Phosphate Transfection Kit (Invitrogen, CA) to generate stable transfectants and protein expression induced in 500 ml cultures with copper sulfate as described by the manufacturer (Invitrogen, CA). The supernatant was filtered using a 0.22-µm filter (Millipore, MA). rP8, rP19 and rP23 were purified from the supernatant by means of the Ni-NTA Superflow column chromatography (Qiagen, CA) and eluted with 250 mM imidazole. The eluted fractions were filtered through a 0.22-µm filter and concentrated with a 5-kDa concentrator (Sigma-Aldrich, MO) by centrifugation at 4°C, washed and dialyzed against PBS. The purity of rP8, rP19 and rP23 was checked by Coomassie blue staining after electrophoreses on SDS 12% polyacrylamide gel and the concentration was determined by BCA protein assay kit (Thermo Fisher Scientific inc., IL).

### Serological analysis by immunoblotting

Equal amounts of purified recombinant salivary proteins (100 ng), were electrophoresed on a SDS 12% polyacrylamide gel and transferred to nitrocellulose membranes. The membranes were blocked with PBS containing 5% milk powder and the immunoblots were probed with a 1∶250 dilution of serum. To demonstrate that sera from immunized rabbits recognize tick salivary proteins, nymphal SGE (2 µg) prepared as described earlier [19] was electrophoresed and blotted as positive control. Immunoreactive bands were visualized using horseradish peroxidase conjugated goat anti-rabbit secondary antibodies (Sigma-Aldrich, MO) and the enhanced chemiluminescence Western Blotting Detection System (GE Healthcare, NJ).

### Detection of glycosylation modifications on recombinant proteins by Periodic acid-Schiffs (PAS) staining

Periodic acid-Schiff (PAS) staining of glycoproteins after electrophoreses of the 3 proteins (25 µg) on SDS 12% polyacrylamide gel [32] was performed with Salp15 as a positive control [51] and BSA as a negative control according to the manufacturer's specifications (Sigma-Aldrich, MO).

### Tick RNA isolation and quantitative RT-PCR

Ticks were fed to repletion on experimental and control animals. Larval ticks were pooled (5 ticks), nymphs and adults were dissected and salivary glands and midguts were pooled (3 ticks), homogenized and RNA was extracted using the RNeasy minikit (Qiagen, CA). The same procedure was done with unfed ticks. cDNA was synthesized using the iScript RT-PCR kit (Biorad, CA) and analyzed by quantitative PCR for the expression of tick actin, *p8, p19, p23* and *p32* and genes using the primers listed in **Table 3**. Quantitative real-time PCR was performed for all tick genes using the iQ Syber Green Supermix (Biorad, CA) on a MJ cycler (MJ Research, CA).

**Table 3:** Primers used.

Target	Forward	Reverse
***Tick actin***	**GGCGACGTAGCAG**	**GGTATCGTGCTCGACTC**
***p8***	**TTACTGCTGGAACGCTGAGA**	**TGGCATTCTCCATTTTGACA**
***p19***	**GAACGAGAGGCAACAGAAGG**	**GCGAGCTTCTTGTTCAGGAT**
***p23***	**TCAACGCTACTTTCGACACG**	**ACACGGTCAGAACCTTGTCC**
***p32***	**TTAGCATACGCCCCCTACAC**	**ACGTTTGAACCCTTTGTTGC**

### Assay for detection of complement-mediated killing of *Borrelia* spirochetes

The serum-sensitive *Borrelia garinii* strain A87S was used (10^7^ spirochetes ml^−1^) to determine complement-mediated killing as described earlier [8]. Spirochetes (2.5×10^5^) were pre-incubated with bovine serum albumin (BSA), Salp15, rP19, rP23 or rP8 (0.24 µg/µl) respectively for 30 min at 33°C. They were then incubated with 12.5% normal human pooled serum (NHS) or heat-inactivated NHS and examined after 1.5 h and 4.5 h. Serum samples were checked for the absence of *Borrelia*-specific antibodies by western blot analysis. Heat inactivation of NHS was performed by incubation at 56°C for 30 minutes. Borreliacidal effect was recorded by screening for immobilization and bleb formation of the spirochetes. Immotile spirochetes were considered dead, as described previously [31]. The percentages of non-viable spirochetes from 200 spirochetes per well were assessed.

### Thrombin generation

Thrombin generation was initiated by recalcification of human pooled normal plasma in the presence of 1 pM recombinant human tissue factor (Innovin, Siemens Healthcare Diagnostics, Germany), 4 µM phospholipids, 417 µM thrombin substrate (z-Gly-Gly-Arg-AMC) and Salp15, rP23, rP8 or rP19 respectively. A calibrated automated thrombogram was used to assay the generation of thrombin in clotting plasma using a Fluoroskan Ascent microtiter plate reading fluorometer (Thermo Fisher Scientific, MA) and Thrombinoscope software (Thrombinoscope BV, Netherlands) according to the manufacturer's instructions. Thrombin formation was followed for 40 min and measurements were taken at 20 second intervals. The endogenous thrombin potential (ETP), lag time and time-to-peak were calculated using the Thrombinoscope software. Experiments were performed in duplicate and repeated three times.

### Immunization of rabbits with recombinant nymphal *I. scapularis* proteins

Rabbits were immunized subcutaneously with 3 doses containing 50 µg of each purified recombinant protein separate, or with a cocktail composed of 30 µg of each recombinant protein, emulsified with Complete Freund's Adjuvant (first dose) and two subsequent booster injections emulsified in Incomplete Freund's Adjuvant at 3-week intervals. Control rabbits were inoculated with adjuvant and OVA (50 µg and 90 µg for the single and cocktail immunizations, respectively). Two weeks after the last immunization, rabbits were infested with 50 *I. scapularis* nymphs on the ear of each rabbit and ticks kept in place using socks over each ear. Nymphs that had fed to repletion and detached were weighed. In an independent control experiment, nymphal ticks were weighed and allowed to molt individually at 23°C and 85% relative humidity under a 14 hour light, 10 hour dark photoperiod.

### Statistical analysis

The significance of the difference between the mean values of the groups was analyzed using the Student *t* test with Prism 5.0 software (GraphPad Software, USA). *p*≤0.05 was considered statistically significant.
